# An evaluation of the content and quality of tinnitus information on websites preferred by General Practitioners

**DOI:** 10.1186/1472-6947-12-70

**Published:** 2012-07-12

**Authors:** Kathryn Fackrell, Derek J Hoare, Sandra Smith, Abby McCormack, Deborah A Hall

**Affiliations:** 1 Division of Psychology, School of Social Sciences, Nottingham Trent University, Burton Street, Nottingham, NG1 4BU, UK; 2 NIHR National Biomedical Research Unit in Hearing, Ropewalk House, 113 The Ropewalk, Nottingham, NG1 5DU, UK; 3 School of Clinical Sciences, The University of Nottingham, Nottingham, UK

**Keywords:** World wide web, Education, Good practice guidelines, Tinnitus management

## Abstract

**Background:**

Tinnitus is a prevalent and complex medical complaint often co-morbid with stress, anxiety, insomnia, depression, and cognitive or communication difficulties. Its chronicity places a major burden on primary and secondary healthcare services. In our recent national survey of General Practitioners (GPs) from across England, many reported that their awareness of tinnitus was limited and as a result were dissatisfied with the service they currently provide. GPs identified 10 online sources of information they currently use in clinical practice, but welcomed further concise and accurate information on tinnitus assessment and management. The purpose of this study was to assess the content, reliability, and quality of the information related to primary care tinnitus assessment and management on these 10 websites.

**Methods:**

Tinnitus related content on each website was assessed using a summative content analysis approach. Reliability and quality of the information was assessed using the DISCERN questionnaire.

**Results:**

Quality of information was rated using the validated DISCERN questionnaire. Significant inter-rater reliability was confirmed by Kendall’s coefficient of concordance (*Wt*) which ranged from 0.48 to 0.92 across websites. The website Map of Medicine achieved the highest overall DISCERN score. However, for information on treatment choice, the British Tinnitus Association was rated best. Content analysis revealed that all websites lacked a number of details relating to either tinnitus assessment or management options.

**Conclusions:**

No single website provides comprehensive information for GPs on tinnitus assessment and management and so GPs may need to refer to more than one if they want to maximise their coverage of the topic. From those preferred by GPs we recommend several specific websites as the current ‘best’ sources. Our findings should guide healthcare website providers to improve the quality and inclusiveness of the information they publish on tinnitus. In the case of one website, our preliminary findings are already doing so. Such developments will in turn help facilitate best practice in primary care.

## Background

The continued growth in use of the World Wide Web as a source of medical information makes it increasingly important that websites deliver health-related content that is up-to-date, reliable and of high quality [1-3]. For tinnitus the need for accurate information is great. There are national guidelines on tinnitus management such as the Department of Health’s Good Practice Guide (GPG) [4], developed by a multidisciplinary team comprising UK-based academics and medico-surgical specialists in neuro-otology, hearing therapy, audiology, General Practice, ENT nursing, clinical psychology, and relevant charities. However, tinnitus is a heterogeneous symptom resulting from or associated with many otological and other medical conditions [5,6]. Added to this, the variability in how patients can respond or not to currently recommended management strategies results in care that regularly relies on clinical experience and opinion rather than a well-defined evidence base [7-10]. This results in a marked lack of standardisation and inequity in the care that different General Practitioners (GPs) currently provide; an issue that the GPG aimed to tackle [4].

Tinnitus is prevalent, affecting about 10% of the population [11]. Tinnitus patients typically access secondary National Health Services (NHS) such as neurotology or audiological rehabilitation through a primary consultation with their GP. Recently, the median number of GP consultations for tinnitus in the UK was reported to be 10–19 within a typical three-month period [12]. We recently surveyed GPs asking them about how they assess and manage these patients and our conclusions highlighted that GPs need concise guidelines to more effectively assess, diagnose, and onward refer their tinnitus patients [7]. Mirroring this, people with hearing-related problems also expressed concerns over poor GP awareness of their needs, appropriate assessment, and referral pathways [13,14], and secondary care providers such as audiologists questioned the appropriateness of tinnitus referrals they receive from GPs [15]. While many GPs agree on the need for improvements to their management of tinnitus, the condition was not considered to sufficiently impact on their practice to warrant dedicated educational workshops [7]. Instead, internet resources were reported to be a favoured medium and GPs identified a number of preferred websites [7]. An advantage of the internet is that it provides instant access to diverse sources of medical information [16]. This is not always helpful however. A simple Google search of ‘tinnitus treatment’ outputs over 11 million results, the first page of which spans private and NHS healthcare sites, self-help options, alternatives to conventional management approaches such as low-level laser therapy, and numerous pages on sound devices, news, and forums. The accuracy, completeness, quality, and reliability of such health information can be compromised, potentially jeopardising rather than facilitating good healthcare provision [2,3,17]. For example, Kieran and colleagues [2] report vast differences in the quality of information between websites, with commercially orientated websites rated as poorer quality.

GPs typically favour specific healthcare sites rather than general search engines as a source of information. As a guide to practice, or educational tools, it is important to establish whether these specific sites provide accurate, reliable, and ‘up to date’ information that will appropriately inform their practice. The internet at large remains unregulated, and websites will consequently vary in the quality and accuracy of their information [16]. One validated tool used to quantify the quality of health information on the internet is the DISCERN questionnaire [18,19].

Here we systematically evaluated the 10 websites reportedly used by GPs as sources of information on tinnitus by (1) assessing their reliability and quality using the DISCERN questionnaire, and (2) evaluating their tinnitus-related content using summative content analysis. Our findings provide recommendations about the current ‘best’ of those preferred websites as practical everyday sources of information and support for GP practice, and recommendations for GP education via this medium in the future.

## Methods

### Selection of websites for evaluation

The selection of websites for evaluation was based entirely on the reports from 368 GPs who took part in a previous study [7] in which we surveyed a random and highly representative sample of GPs from across England. Eighteen percent of those GPs reported having a particular interest in ENT.

The initial set of 14 websites comprised Map of Medicine [20], the British Tinnitus Association (BTA) [21], GP notebook [22], eMedicine [23] (now Medscape), Clinical Knowledge Summaries (CKS) [24], EMIS Mentor/Web Mentor (by EMIS, Egton Medical Information Systems) [25], GP online [26], Royal National Institute for Deaf people (now Action on Hearing Loss) [27], patient.co.uk [28], NHS Choices [29], National Institute for health and Clinical Excellence (NICE) [30], doctors.net [31], and GP mentor [32]. The websites selected for analysis in the present paper represent all those that were identified by GPs and that could be considered as direct providers of health information (i.e. not search engines or collections of links). The above set of websites was therefore reduced by four for the following specific reasons. First, Web Mentor and EMIS Mentor were found to be two different references to the same website (mentor-online.com [25]) and hence were reduced to one entry. Second, the GP mentor website was designed primarily as a revision tool for medical students and GP exams. Third, NICE did not contain any specific information on tinnitus. Finally, doctors.net was also excluded as it was found not to provide information specific to tinnitus, but rather provides a forum space, email accounts, and a ‘library’ style repository linking to medical journal websites. The dates over which each website was assessed are given in Table 1.

**Table 1:** Websites and assessment dates

**Website**	**Dates assessed**
Map of Medicine [20]	19-21 July 2011
BTA [21]	14 July −3 Aug 2011
GP notebook [22]	20 July −29 Aug 2011
eMedicine [23]	22-26 July 2011
CKS [24]	22-27 July 2011
Mentor-online [25]	20-25 July 2011
GP online [26]	28 July – 1Aug 2011
Action on Hearing Loss [27]	18 July - 4Aug 2011
patient.co.uk [28]	18 July - 4Aug 2011
NHS Choices [29]	18-21 July 2011
NICE [30]	19-20 July 2011
Doctors.net [31]	27-28 July 2011 and 12–13 June 2012
GP Mentor [32]	12-25 July 2011

*BTA* British Tinnitus Association, *CKS* Clinical Knowledge Summaries. NICE National Institute for health and Clinical Excellence.

### Website details

A paper-based form was developed to systematically extract data from each website including general descriptive details, and tinnitus-related content. Development of this form is described below. Website details extracted included the intended purpose (e.g. whether the site promotes alternative therapy or self-help), producer of the website (e.g. charity, commercial venture, NHS), the primary target audience of the website (patients, healthcare workers), and the usability of each site (search function, ease of finding a particular piece of information). Details of accreditations held by each website were also recorded.

### Reliability and quality assessment: the DISCERN questionnaire

This DISCERN questionnaire is used to evaluate reliability, quality, and trustworthiness of general and treatment-specific healthcare information. DISCERN highlights where there are gaps in information, such as information relating to patient support or to alternative therapies, and high DISCERN scores are associated with those websites which tend to have quality kite-marks such as independent accreditations [1,16]. DISCERN was developed through an iterative process involving clinical specialists, self-help group representatives, GPs, a consumer health information expert, a healthcare consumer, a lay medical publisher, a medical journalist, and representatives from the Plain English Campaign, the Community Health Council, and the NHS Centre for reviews and Dissemination [19]. The questionnaire was subsequently tested for validity on UK sample groups of health information providers and members of various self-help groups, and was found to show significant levels of agreement on quality scores across groups (chance corrected agreement kappa = 0.56) and to have good face and content validity [18].

Sixteen questions (Appendix 1) contribute to a 5-point Likert scale (score 1 = No, score 5 = Yes), and it is the *combined* scores of multiple questions that is of particular interest [33]. Intermediate ratings of 2–4 indicate that the website met that criterion to some degree (i.e. partially). The DISCERN handbook [19] provides clear examples to aid rating on each question. For example, Question 8 asks whether the information being assessed contains details of treatment uncertainties. To score 5 points (Yes) there must be clear reference to any uncertainty regarding tinnitus treatment choices: this may be linked to each treatment choice or may be covered in a more general discussion or summary of the choices mentioned. To score 2–4 points (partially) treatment uncertainty must be mentioned but the information is then judged either unclear or incomplete to different degrees. A score of 1 (No) would mean that no uncertainty about tinnitus treatment choice is mentioned in the information being assessed.

The questionnaire is separated into three sections. Section 1 (questions 1 – 8) addresses the general reliability and trustworthiness of the website. For example, reliability is measured by asking whether the sources of evidence are explicit, and assessing common causes of inaccurate or unreliable information such as whether the publication or the information on which it is based is out of date, whether there is evidence of bias, or whether the information fails to refer to a certain treatment option [18].

Section 2 (questions 9 – 15) focuses on quality and detail of information related to treatment choices. Section 3 (question 16) asks for a single overall quality rating of the resource as a source of information about treatment choice based on all 15 preceding questions. We chose to answer Question 16 as an average of scores for questions 1–15.

Sets of three authors evaluated each website independently. To assess the level of agreement between raters of the same website, Kendall’s coefficient of concordance [34] was calculated for DISCERN scores (weighted for ties, *Wt*). Calculations were performed in *R* (Version 2.14.0). Kendall's *W* ranges from 0 (no agreement) to 1 (complete agreement), with values of 0.40 to 0.80 generally considered to indicate moderate (acceptable) agreement. After DISCERN scores were awarded individually by each rater, all three raters assessing the same website met to discuss and reach a consensus on final scores for each question in turn.

### Summative content analysis

#### Tool development

We chose to conduct a summative content analysis of the text available on each website as this method of content analysis is used successfully and increasingly in health research (see [35] for more details). This approach incorporates latent content analysis which considers the *context* in which particular words are used, i.e. it *qualifies* word use, as well as *quantifies* word use as in manifest content analysis [36,37]. Development of the paper tool, to extract data for content analysis, was guided by Petch [38] and the NHS commissioning guidance published in the Department of Health GPG [4].

In step 1 of tool development a pilot form was created with sections to record (1) general website detail (intended purpose, source, target audience, functionality, details of any accreditations, and any website links) and (2) the use and context of use of specific keywords or phrases related to the assessment and management of tinnitus. The selection of keywords and phrases was derived from the GPG [4], covering standard tinnitus patient assessments, referral criteria, ‘red flags’ for emergency referrals, and recommended GP management strategies. All terminology relating to primary care in the ‘Suggested components of the Tinnitus network’ section of the GPG was selected as keywords. Note that this paper does not evaluate the chosen websites against *all* the keywords in the GPG, but only those keywords relating to primary care. Thus for example, management strategies such as cognitive behavioural therapy fall outside the scope of GP practice. The pilot tool also left free space to record any additional referral criteria or red flags not identified in the GPG, and the levels of supporting evidence used by each websites (e.g. expert opinion, original research articles, or systematic reviews).

Step 2 involved piloting the data extraction tool on three websites that would not be included in the final analysis. None of the ‘pilot’ websites had been cited by GP’s in our previous study [4], but were nevertheless relevant to tinnitus. These were websites of the American Tinnitus Association [39], The Tinnitus Clinic [40], and Deafness Research UK [41], all accessed between 4^th^ and 8^th^ July 2011. Authors independently accessed each website recording all details on the pilot form. The aim of conducting a pilot was to assess usability and comprehensiveness of the tool, and identify any redundant sections. As a collective, all authors thereafter met to refine the tool and agree on a second and final iteration for use in the study. The keywords and phrases relating to assessment and management are given in Tables 2 and 3 respectively.

**Table 2:** Content analysis: information on tinnitus assessment

**Website**	**Sudden hearing loss¶**	**Significant distress¶**	**Cranial nerve symptoms¶\**	**Pulsatile tinnitus¶**	**Unilateral hearing loss ®**	**Persistent tinnitus ®**	**Unilateral tinnitus ®**	**Impact on QOL ®**	**Anxiety**	**Depression**	**Sleep problems**	**Onset, causes**	**Tinnitus pitch/loudness**	**Hearing loss**	**Hearing difficulties**	**Otoscopy**	**Carotid bruit**	**General medical**
**Map of Medicine**	**√√**	**√√**	√	**√√**	**√√**	√	**√√**	√	√	√	√	√	√	√	√	√	√	√
**BTA**				√				√	√	√	√	√	√	√	√	√		√
**GP notebook**	**√√**	**√√**	√	**√√**	**√√**		**√√**	**√√**	√	√	√	√	√	√	√	√	√	√
**eMedicine**				√	√		√	√	√	√	√	√	√	√		√	√	√
**CKS**	**√√**	**√√**	√	√	**√√**		**√√**	**√√**	√	√	√	√	√	√		√		√
**Mentor-online**				√		√	**√√**	√	√	√		√	√	√		√	√	
**GP online**		**√√**	√	**√√**		√	**√√**	√	√	√	√	√	√	√	√			√
**Action on Hearing Loss**	**√√**	√		√	**√√**		**√√**	√	√	√	√	√	√	√	√	√	√	√
**Patient.co.uk**			√	√		√	**√√**	√	√	√	√	√	√	√	√	√	√	√
**NHS Choices**				√			√	√	√	√	√	√	√	√				√

Use of each keyword or phrase on each website is indicated by a single tick (√). A double tick (√√) indicated that the keyword or phrase was used in the context of a referral criterion as defined by Department of Health. A blank cell indicates the keyword or phrase was not used. Sudden hearing loss, significant distress, cranial nerve symptoms and pulsatile tinnitus are all defined as ‘red flags’ for immediate referral to secondary care (¶). Unilateral hearing loss, persistent tinnitus, unilateral tinnitus, and impact of Quality of Life (QOL) are defined as criteria for referral to secondary care ®. *BTA* British Tinnitus Association, *CKS* Clinical Knowledge Summaries.

**Table 3:** Content analysis: information on tinnitus management options

**Website**	**Ear wax removal**	**Ear infection**	**Blood pressure**	**Information leaflets**	**Educate patient**	**Information on self-help**	**Sound devices**	**Hearing aids**	**Self-help/ groups**	**Anti-depressants**	**Anxiolytics**	**Night sedation**
**Map of Medicine**	√	√		√	√	√	√	√	√			
**BTA**	√	√	√	√	√	√	√	√	√	√	√	√
**GP notebook**	√	√	√	√	√	√	√	√	√	√		√
**eMedicine**	√	√					√	√	√	√	√	
**CKS**	√	√		√	√	√	√	√	√	√		√
**Mentor-online**	√	√		√	√	√	√	√	√	√		
**GP online**	√	√			√	√	√	√	√	√	√	√
**Action on Hearing Loss**	√	√	√	√		√	√	√	√	√	√	√
**Patient.co.uk**	√	√	√	√	√	√	√	√	√	√		
**NHS Choices**	√	√	√			√	√	√	√	√		

Use of each management keyword or term on each website is indicated by a single tick (√), as defined by the Department of Health Good Practice Guide. *BTA* British Tinnitus Association, *CKS* Clinical Knowledge Summaries.

#### Conduct of content analysis

Summative content analysis of each website was carried out independently by sets of three of the five authors between 12^th^ July and 29^th^ August 2011. The allocation of authors to each website was unbiased because it was decided using an electronic random number generator. Authors first independently accessed each website and recorded relevant details using the data extraction tool, searching for website details and noting the occurrence of target key words or phrases. Having identified key words, authors then recorded the context in which they were used, e.g. “mentioned as a management option”, or, “defined as an indicator of potential medical emergency”. The three authors assessing the same website then met again to reach a consensus of the data extracted for each section of the form. Finally, all five authors met to review the extracted data and ensure that each field within the data extraction tool had been interpreted in a consistent manner. Data was recorded in Microsoft Excel.

## Results

### Website details

A summary of website details is given in Table 4. Six of the 10 websites were commercially produced, two were charity websites, and two were government produced. All 10 websites provided information that is targeted at clinicians either as the main target audience (n = 6), or through a specific section of the site targeting health professionals (n = 4). Of the 10 websites, only three (BTA, Action on Hearing Loss, and NHS Choices) were judged to contain information on health promotion. Three posted recent news on tinnitus, and referred to alternative or complementary therapy (BTA, Action on Hearing Loss, and eMedicine). All websites were found to contain information on self-help.

**Table 4:** Details of 10 healthcare websites that were assessed in this study

**Website**	**Intended purpose**	**Produced by**	**Targets clinicians?**	**Functionality**	**Accreditation**	**Links to..**
**Map** **of** **Medicine**[20]	Provides care maps used to establish new services, manage referrals and communicate across care settings.	Commercial, Hearst Business Media.	Yes, for GPs and secondary health care.	Homepage confusing but maps themselves easy to navigate.	The Information Standard.	AOHL, BTA, CKS, PAT. DH GPG.
**British** **Tinnitus** **Association**[21]	Provides support and information for people with tinnitus.	Registered charity.	Yes, dedicated section.	Easy navigate, clearly marked, menu bar helpful. However lots of scrolling needed, and cannot use the ‘back’ button.	The Information Standard.	AOHL.
**GP Notebook**[22]	Provides encyclopaedic synopses focussed on the needs of GPs.	Commercial Oxbridge Solutions Ltd.	Yes, GPs.	Easy use, reliable search function. Required the use of back button to get to additional areas from the first page.	None	BTA. DH GPG.
**eMedicine**[23]	To provide healthcare professionals with information to improve patient care and stay current in their practice.	Commercial - WebMD Health Professional Network.	Yes, general `healthcare.	Overly burdened with text, with few subsections, heading or summaries, making it very laborious to find particular information.	None	None
**Clinical** **Knowledge** **Summaries**[24]	Aims to provide evidence-based information and practical ‘know how’ on conditions typically managed in primary care.	Government – NHS.	Yes, aimed at clinicians working in Primary and first contact care.	Easy to navigate, logical drop-downs and lists. Can get to any required information in 2 – 3 drills.	Approved by Plain English Campaign.	None
**Mentor** **Online**[25]	Aims to provide evidence-based information for medical decision-making.	Commercial - Egton Medical Information Systems Ltd (EMIS).	Yes, medical practitioners.	Well structured but requires practice to navigate. Useful shortcuts.	None	AoHL, BTA, CKS, GPN, NHS C, PAT.
**GP Online**[26]	Aims to provide GPs with news, clinical and management resources, focused CPD and educational materials.	Commercial, Haymarket Business Media.	Yes, GPs.	Site is difficult to navigate, individual pages are very long, but related information not always on the same page and difficult to find.	None	AOHL, BTA, CKS, DH GPG.
**Action** **On** **Hearing** **Loss**[27]	Aims to support people with hearing loss or tinnitus, and promote hearing health.	Registered charity.	Yes, section for GPs.	Easy to navigate but GP section is ‘buried’ in the ‘S*upporting you’* section. Search function not useful as invariably directs you to forum pages.	None	BTA
**Patient.co.uk**[28]	Provides non-medical people with information about health and disease.	Commercial - Egton Medical Information Systems Ltd (EMIS)	Suggests health professionals may also find the site a useful resource.	Clear layout, but too much information on main pages. Good use of sections and tabs.	The Information Standard. HONcode standard for trustworthy health.	AOHL, BTA, CKS, GPN, NHS C, EMED.
**NHS Choices**[29]	Aims to provide information on self care, from lifestyle decisions to practical aspects of using NHS services.	Government - NHS	Contains a section for healthcare professionals but not related to medical practice.	Easy to navigate but excessive text. Some very user friendly search features.	The Information Standard.	AOHL, BTA, CKS, MOM.

*AOHL* Action on Hearing Loss, *BTA* British Tinnitus Association, *CKS* Clinical Knowledge Summaries, *DH GPG* Department of Health Good Practice Guide, *EMED* eMedicine, *GPN* GP Notebook, *MOM* Map of Medicine, *NHS-C* NHS Choices, *PAT* patient.co.uk.

All 10 websites had a search function that was generally easy to find. There were however, differences in its usability, and in the relevance of search results. For example, for CKS a search of ‘tinnitus’ generated different results depending on which website page the search was initiated. For mentor-online, results were limited when searching ‘tinnitus treatment’ or ‘tinnitus assessment’ in that only the first word (tinnitus) was used in the search and subsequent words (assessment or treatment) were ignored. GP notebook’s search function also ignored multiple-word search terms, searching for each word individually, i.e. there was no way of limiting a search to specific aspects of tinnitus. In contrast, Map of Medicine effectively filtered results in real time as the search term was entered and this enabled the user to narrow down the search to tinnitus assessment very effectively.

Functionality further varied across websites. Although on all websites the ‘tinnitus’ page was easily found, the amount of information and the manner of its presentation varied greatly. The BTA site was easy to navigate, but there was a lot of repetition of information across leaflets and web pages. eMedicine had a particularly large volume of information to read through. GP online, mentor-online, and Action on Hearing Loss were all judged to spread related information (e.g. referral criteria) across various pages, requiring substantial amounts of time to go between pages to piece together a more comprehensive picture. In contrast, Map of Medicine plots out a clear referral pathway incorporating all such information on a single page, minimising the time required to collate the information (discussed later).

Websites were checked for formal independent accreditations. Four (BTA, Map of Medicine, NHS Choices, and patient.co.uk) were accredited with The Information Standard. One website, CKS, was approved by the Plain English Campaign.

### Quality assessment: The DISCERN questionnaire

Part of the DISCERN questionnaire assessed the quality of information on tinnitus posted on each site. Interestingly, only one website, CKS, described the search strategy they had used to source the information presented. All other websites failed to explicitly cite their sources. Only five websites (Map of Medicine, eMedicine, CKS, Mentor-online, and patient.co.uk) used any form of academic referencing, and of those, only patient.co.uk referred to the Cochrane database, a rich source of systematically reviewed information for making decisions on questions in healthcare [42].

DISCERN scores (Section 1, Section 2, and overall score), and inter-rater reliability of scores for each website are shown in Table 5. Websites are listed in rank order of overall average score. Inter-rater reliability (Kendall’s *Wt*) for all 10 websites was statistically significant, with most ratings falling in the category of moderate agreement (i.e. range = 0.40 - 0.80).

**Table 5:** DISCERN Questionnaire scores

**Website**	**Used by% GPs**	**Section 1 score**	**Section 2 score**	**Overall score**	**Kendall's coefficient of concordance**
**Map of Medicine**	2	4.6	2.3	3.5	*Wt* = 0.85, *df* = 2, *p* = 0.001
**BTA**	2	3.3	3.1	3.2	*Wt* = 0.67, *df* = 2, *p* = 0.014
**GP notebook**	52	3.5	2.4	3.1	*Wt* = 0.84, *df* = 2, *p* = 0.001
**eMedicine**	1	3.6	2.6	3.1	*Wt* = 0.45, *df* = 2, *p* = 0.002
**CKS**	7	3.9	1.9	2.9	*Wt* = 0.84, *df* = 2, *p* = 0.001
**Mentor-online**	15	3.9	1.6	2.8	*Wt* = 0.82, *df* = 2, *p* = 0.002
**GP online**	<1	3.4	2.1	2.8	*Wt* = 0.76, *df* = 2, *p* = 0.004
**Action on Hearing Loss**	2	2.8	2.6	2.7	*Wt* = 0.79, *df* = 2, *p* = 0.003
**Patient.co.uk**	19	4.0	1.6	2.6	*Wt* = 0.92, *df* = 2, *p* < 0.001
**NHS Choices**	<1	2.8	2.0	2.5	*Wt* = 0.75, *df* = 2, *p* = 0.005

Section1, Section 2 and Overall score for each website are presented as mean of all questions (8 questions in Section 1, 7 questions in Section 2, 15 questions overall). Values are averages corrected to one decimal place. Websites are listed in rank order of overall score. Scores can range 0 to 5 where a score less than 5 suggests some ‘potentially important but not serious shortcomings’ and a score of less than 3 suggests some ‘potentially serious shortcomings’ in the information. BTA = British Tinnitus Association, *CKS* Clinical Knowledge Summaries. Inter-rater reliability is reported as Kendall’s *Wt*. ‘Used by% GPs’ refers to the proportional use of each website by GPs in our previous study [6].

#### DISCERN SECTION 1: Reliability of the information

Map of Medicine achieved high scores for all questions in Section 1 scoring an average of 4.6 out of 5, indicating that it had ‘minimal shortcomings’ in terms of the general reliability of the information provided. All other websites scored above 3 with the exception of NHS Choices which scored an average of 2.9, and Action on Hearing Loss, which scored 2.8. Scores below 3 suggest some ‘potentially serious shortcomings’ in the information (although scoring is to some extent down to personal judgement, the DISCERN handbook [19] provides examples and guidelines for rating every individual question).

All 10 websites achieved high scores (≥3) for the clarity of their aims, and for providing additional sources of support and information, e.g. links to other websites. However, scores were more variable for questions relating to details of the evidence base or sources of information used (Appendix 1, Questions 4 and 5). Five websites achieve a score of 4 or above for these questions (CKS, eMedicine, GP notebook, Map of Medicine, and Mentor-online), but Action on Hearing Loss only scored 1 for Question 4, and 2 for Question 5. Scores for NHS Choices, the BTA and GP-online also fell below 3 for these questions. In short these latter four websites provided little or no detail on their sources of information, or how up-to-date the information they provided was.

#### DISCERN SECTION 2: Quality of the information on treatment choices

The BTA scored highest for Section 2 (3.1 out of 5) indicating ‘some important but not serious shortcomings’ in information on treatment choices. All other websites were scored below 3 indicating ‘potentially serious shortcomings’ in this information. For Section 2, scores for the individual questions were generally low. Most websites never achieved higher than a score of 3, with the possible exception of Question 14 (Appendix 1) which asked if there was information about more than one treatment choice, for which all websites achieve a 3 or above, simply because all sites discussed to some extent a number of treatment options. Of note, the BTA, GP notebook, and eMedicine all scored the maximum 5 for Question 14. In contrast, all websites except the BTA (which scored 3), scored the minimum (1) for failing to describe what would happen if no treatment was used (DISCERN Question 11, Appendix 1). The risks associated with different treatment (Question 10, Appendix 1) were also poorly represented on most websites, with the BTA and eMedicine scoring highest (3) on this question. Only Action on Hearing Loss, the BTA, and Map of Medicine suggest discussing treatment choices with family and involving patients in shared decision making (Question 15, Appendix 1).

#### DISCERN SECTION 3: Overall quality scores

From the overall ranking of the average scores (Table 5), Map of Medicine came out on top with 3.5 out of a possible 5. This score indicates overall ‘some potentially important but not serious shortcomings’, these tending to be related to the quality of the information on treatment choices rather than the reliability of the information provided. NHS Choices achieved the lowest overall score (2.5) and was rated as having ‘potentially serious shortcomings’ in its information on tinnitus, in terms of both quality and reliability.

### Summative content analysis

A summative content analysis approach was used to assess the usage and context of use for keywords and phrases related to tinnitus assessment and management cited in the GPG for tinnitus [4]. Tables 2 and 3 summarise whether keyword were identified (denoted √) or not (denoted by a blank cell). No gross error or misinformation was found. The average time per researcher spent extracting data from each website was also recorded (Table 6), providing some indication of the resource a GP would need to expend to gather a subset of information on tinnitus from each site, if they were naive to the site. The average time taken per researcher to extract the required data from each site was 43 minutes (range average 35–60 minutes).

**Table 6:** Average time per researcher spent extracting data from each website

**Website**	**Minutes (SD)**
**Map of Medicine**	35 (0)
**BTA**	50 (17)
**GP notebook**	40 (5)
**eMedicine**	55 (5)
**CKS**	50 (5)
**Mentor-online**	35 (9)
**GP online**	50 (10)
**Action on Hearing Loss**	35 (9)
**Patient.co.uk**	45 (5)
**NHS Choice**	35 (0)

Section1, Section 2 and Overall score for each website are presented as mean of all questions (8 questions in Section 1, 7 questions in Section 2, 15 questions overall). Values are averages corrected to one decimal place. Websites are listed in rank order of overall score. Scores can range 0 to 5 where a score less than 5 suggests some ‘potentially important but not serious shortcomings’ and a score of less than 3 suggests some ‘potentially serious shortcomings’ in the information. BTA = British Tinnitus Association, CKS = Clinical Knowledge Summaries.

#### Tinnitus assessment

In Table 2, desirable use of the keyword or phrase in the context of an urgent or routine referral criterion, as defined by the GPG, is denoted “√√”. Assessment results that indicate a need for urgent referral to secondary care include sudden hearing loss, significant distress, cranial nerve symptoms, or pulsatile tinnitus, while more routine referral criteria relate to unilateral hearing loss, persistent tinnitus, unilateral tinnitus, or significant impairment of quality of life (QOL). The GPG further recommends the assessment of anxiety, depression, insomnia, tinnitus onset, etiology, pitch, loudness, hearing loss (physiological), difficulties hearing (functional), carotid bruit, as well as a general medical assessment and otoscopy. Of the 10 websites assessed only Map of Medicine mentioned all of these assessment criteria. This is reflected in its top ranking on overall DISCERN score. Map of Medicine was therefore rated both comprehensive and reliable. However, this website identified only 5 of the 8 keywords in the context of referral criteria. Overall, GP notebook (ranked third on DISCERN) reported most keywords in the context of their being referral criteria (6 of the 8). However it failed to mention persistent tinnitus and only mentioned cranial nerve symptoms in relation to a recommended examination. Of the 10 websites assessed, only four (Map of Medicine, mentor online, GP online, and patient.co.uk) mentioned persistent tinnitus, and only five (Map of Medicine, GP notebook, CKS, GP online, and patient.co.uk) mentioned cranial nerve symptoms, but never in the context of it being a criterion for referral to secondary care. Pulsatile tinnitus was referred to on all 10 websites, but only three (Map of Medicine, GP notebook, and GP online) referred to it in the context of being a criterion for referral to secondary care. The impact of tinnitus on QOL was also mentioned on every website, but only two websites (GP notebook and CKS) described it as a criterion for referral. Two websites (BTA, and NHS Choices) failed to refer to any keywords in the context of referral criteria.

Assessment of anxiety, depression, onset, causes, tinnitus pitch, loudness, and hearing loss were found on all 10 websites. Assessment of hearing difficulties or carotid bruit was only identified within six websites (Table 2). GP notebook, Action on Hearing Loss, and patient.co.uk mention all additional assessments, whilst NHS Choices did not mention hearing difficulties, carotid bruit, or otoscopy. That NHS Choices failed to mention these important keywords is reflective of its rating as lowest on overall DISCERN score.

#### Tinnitus management

Management options include ear wax removal, treatment of ear infection or high blood pressure, providing information leaflets, patient education, information on self-help, sound devices, hearing aids, self-help groups, anti-depressants, anxiolytics, or night sedation. Table 3 provides results of the content analysis for keywords and phrases related to these management options (√ denotes use).

Only the BTA website mentioned all management keywords, consistent with its highest ranking for DISCERN Section 2 score. In fact, BTA was the only website to score above 3 for this section. However, most websites did mention the majority of management options listed on the data extraction form. All websites identified ear wax and ear infection as conditions that are sometimes associated with tinnitus [4,6,43] and that should be assessed and treated as appropriate. All websites refer to sound devices, hearing aids, and self-help as possible treatment options. Management of high-blood pressure [5] was mentioned least (by 6 of 10 websites). CKS, mentor-online, GPonline and eMedicine did not mention it. Although most websites discussed anti-depressants, only half referred to night sedation (5 of 10 websites) for the relief of co-morbid insomnia, and less than half mentioned anxiolytics (4 of 10). Surprisingly, given its comprehensive coverage of other aspects of tinnitus, Map of Medicine was the only website not to provide information on any of the three main classes of medication recommended for the relief of tinnitus-related distress or insomnia.

## Discussion

This is the first systematic analysis of websites as sources of information on tinnitus that uses the DISCERN questionnaire, and the first to assess a comprehensive list of websites specifically used as such by medical professionals. GPs need to access information that is presented in a succinct and accessible manner. Here we systematically evaluated reliability, quality and content of the 10 websites identified by GPs in England as their primary sources of information on tinnitus [7]. Our methods used the validated DISCERN questionnaire and summative content analysis. Our results provide a concise summary of website content related to tinnitus assessment and management as set out in the Department of Health’s GPG.

Overall DISCERN quality scores of most websites were limited by a number of factors. For example, detail of how the information presented was gathered and collated was scant, with the exception of CKS which detailed a systematic search strategy. Map of Medicine ranked highest on its overall DISCERN score and hence seems to be the best, most reliable website for information on tinnitus. It was the only website to mention all GPG recommended assessment criteria as well as most of the recommended management options. All the information was easily accessible and it presented a clear procedure for GPs to follow when deciding whether or not to refer tinnitus patients to secondary care. However, the website achieved a low DISCERN Section 2 score suggesting that there was little depth of information on all relevant management choices. Interesting to note that despite our positive evaluation it does not appear to be widely used in primary care. Our previous survey indicates that only 2% of GPs reported using Map of Medicine. The BTA website ranked second on overall DISCERN score, but had the highest score for Section 2 hence was judged the best source of information on management choice. This website had some limitations too however. Information was found to be repeated on different sections of the website, and the website failed to refer to important referral criteria. Again, our previous survey indicated that only 2% of GPs access the BTA website in their clinical practice. Of note, both charity websites (BTA, Action on Hearing Loss) scored highest for DISCERN Section 2, suggesting more depth to the information they provide on management choices. However, the main audience for these two websites is the general public and not healthcare professionals. Our findings indicate that a minimum of 2 websites (e.g. Map of Medicine and BTA) would need to be visited to gather all necessary information recommended by the Department of Health for good practice in tinnitus care. This is highly unlikely to happen in a busy general practice. The results of this systematic analysis can be used by website providers to revise their content to be comprehensive.

Previous studies on health information on the internet using the DISCERN questionnaire considered the value of information from the perspective of the patient [1,16]. The only previous study evaluating information on tinnitus on the internet did not apply the DISCERN questionnaire and excluded any information targeted at clinicians, restricting analysis to information targeting tinnitus patients [2]. All of these studies mentioned above similarly report that health information on websites varies in quality, and recommend that websites should attain independent accreditations. Within the present study, five websites were found to be independently accredited. Accreditation is typically associated with higher levels of trustworthiness, and reliability (and thereby higher DISCERN scores), and previous research has found that patients and care providers are more likely to search those sites with accreditations [2,16]. The association between accreditation and higher DISCERN scores does not hold strictly true for our data however. Both our highest and lowest ranked websites (Map of Medicine and NHS Choices, respectively) have been accredited by The Information Standard. It is likely that a more general assessment of website quality (not restricted to information on a single medical condition as we have done here) would yield different results that may reflect the value of achieving independent accreditation. It is recommended therefore that all websites seek accreditations.

GPs are unlikely to use websites which require a great deal of search and reading time. From our previous work we know that GPs in England have a clear preference for GP notebook (preferred by 52% of those responding to our survey) over other websites [7]. In the present study, this website took an average of 40 minutes research to extract the data required for content analysis. In contrast, Map of Medicine is preferred by only 2% of GPs. This website required a similar amount of research time (35 minutes) to extract data. This, coupled with the higher DISCERN scores for Map of Medicine suggest it is a more comprehensive and more reliable website in terms of information on tinnitus, and perhaps also more user friendly than that currently preferred by most GPs.

## Conclusion

Here we examined the content and quality of information on tinnitus on all those websites currently used by GPs in England finding that no single website provided a comprehensive source of information on tinnitus. The best rated websites appeared to provide either acceptable levels of information on tinnitus assessment *or* management. At present therefore it is advisable that GPs extend their use of internet sources to more than a single website if they are to adhere to Department of Health guidelines, or perhaps more simply, use the GPG as their guide. We recommend the websites Map of Medicine and the BTA as current ‘best’ sources but make specific recommendations for future improvement. This research should serve as a tool for the providers of these websites to reassess the information they provide and consider revisions that will make for more comprehensive coverage of tinnitus assessment and management that will inform best practice and promote best patient care. Indeed, Action on Hearing Loss has already gathered our preliminary findings on this project in preparation for the forthcoming revision of their website which will incorporate some of the recommendations made here.

## Appendix 1 DISCERN questionnaire

SECTION 1: Is the information reliable?

1. Are the aims clear?

2. Does it achieve its aims?

3. Is it relevant?

4. Is it clear what sources of information were used to compile the website?

5. Is it clear when the information used or reported in the publication was produced?

6. Is it balanced and unbiased?

7. Does it provide details of additional sources of support and information?

8. Does it refer to areas of uncertainty?

SECTION 2: How good is the quality of information on treatment choices?

9. Does it describe how each treatment works?

10. Does it describe the benefits of each treatment?

11. Does it describe the risks of each treatment?

12. Does it describe what would happen if no treatment is used?

13. Does it describe how the treatment choices affect overall quality of life?

14. Is it clear that there may be more than one possible treatment choice?

15. Does it provide support for shared decision-making?

16. Based on the answers to all of the above questions, rate the overall quality of the publication as a source of information about treatment choices

## Competing interests

DJH and DAH are serving members of the British Tinnitus Association, on the Professional Advisory Committee and the Board of Trustees, respectively. KF received a student bursary from Action on Hearing Loss to take part in this project.

## Authors’ contributions

KF acquired, analysed and interpreted data, coordinated the study, and drafted the manuscript, DJH conceived of the study, participated in the design, acquired, analysed and interpreted data, and helped draft the manuscript, SS and AM acquired, analysed and interpreted data, DAH participated in design, acquired, analysed and interpreted data, and edited the manuscript. All authors approved the final manuscript. DJH (DipN, PhD) and DAH (PhD) have substantial expertise in tinnitus and have published several recent studies using qualitative research methods and evaluations of the GPG in primary and secondary care. DJH has 10 years clinical experience working in NHS. KF is a final-year psychology student with training in qualitative research methods. SS (BSc) is experienced in tinnitus research and qualitative analysis. AM (PhD) has experience in tinnitus research and significant expertise in qualitative website analysis. All authors read and approved the final manuscript.

## Pre-publication history

The pre-publication history for this paper can be accessed here:

http://www.biomedcentral.com/1472-6947/12/70/prepub
